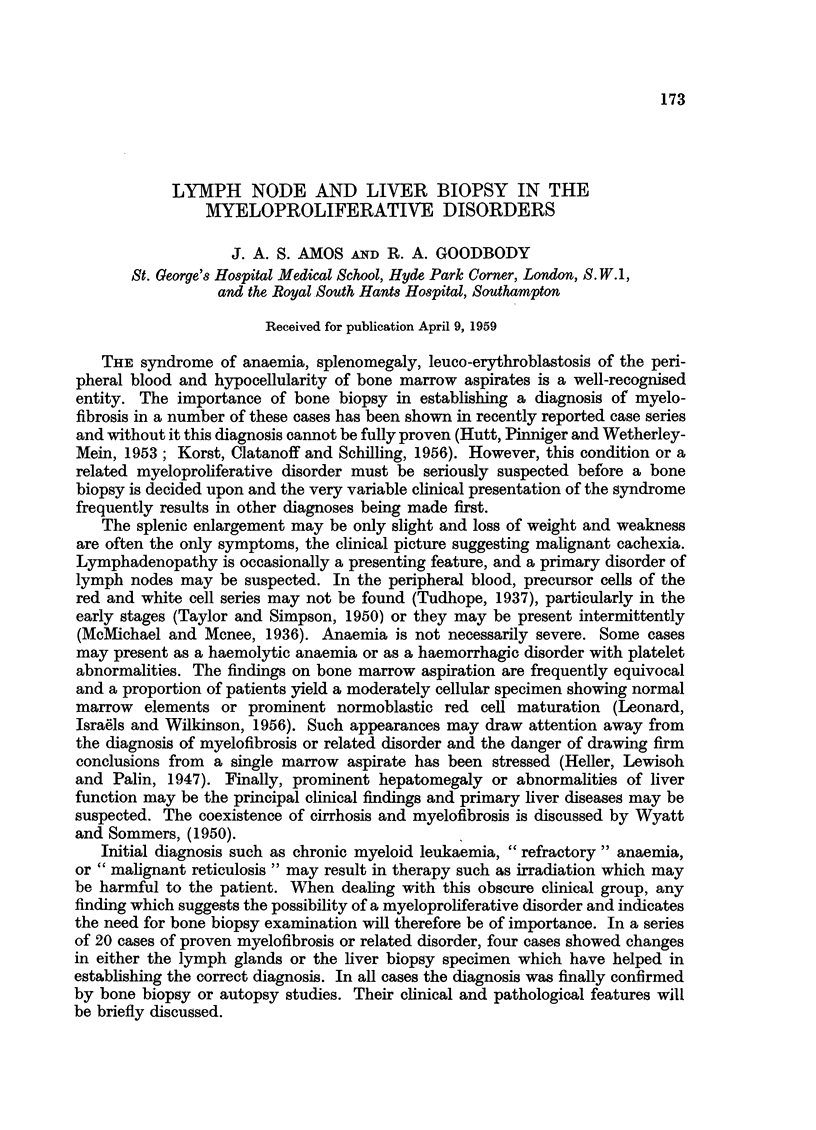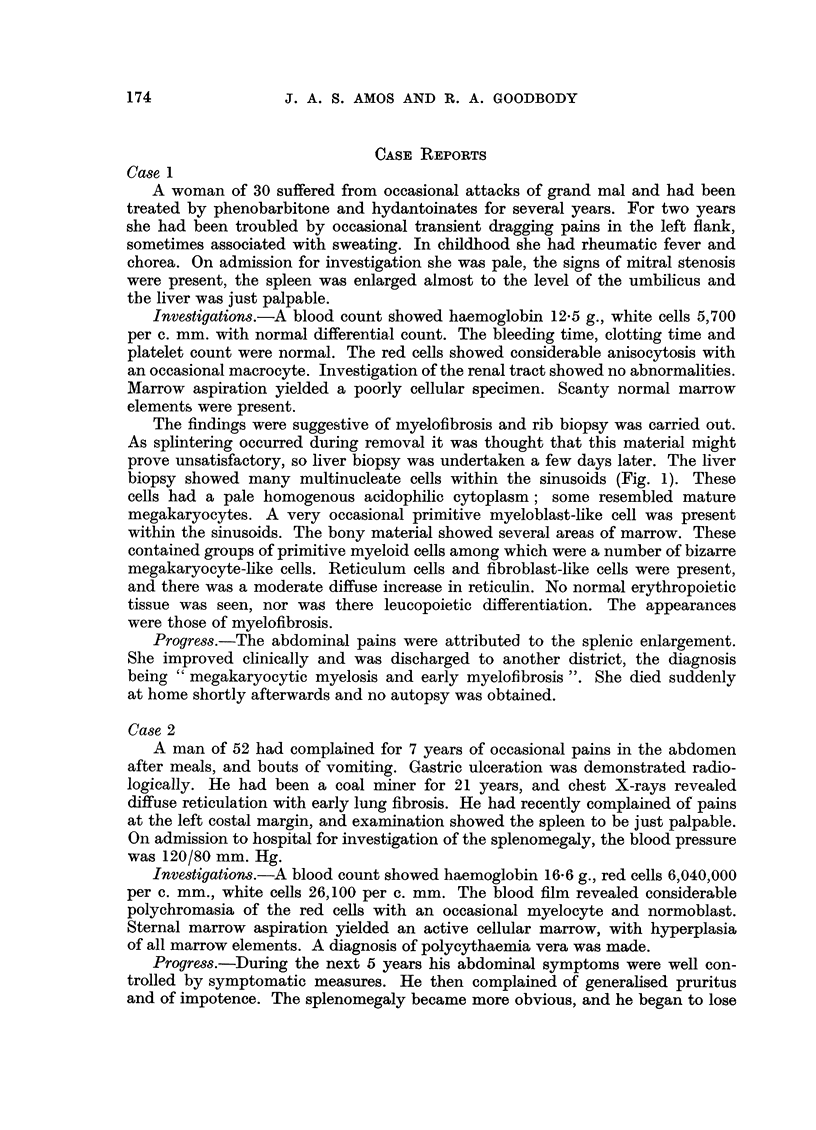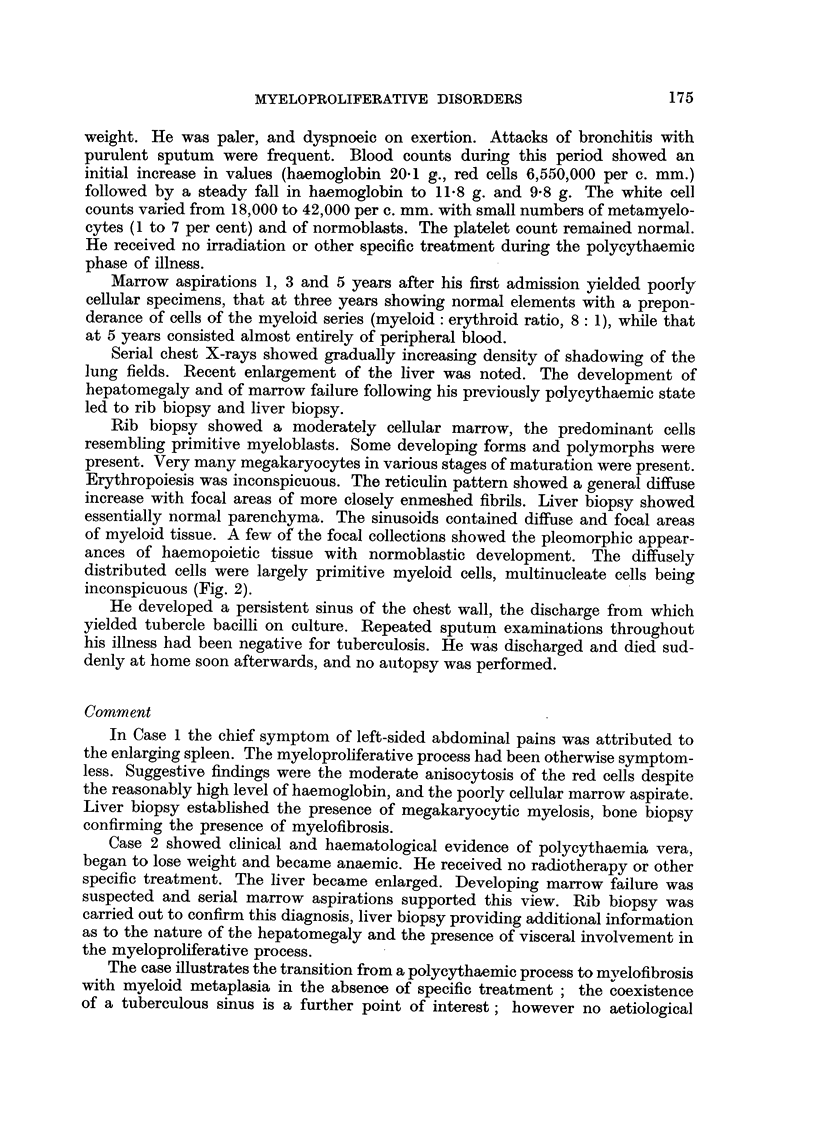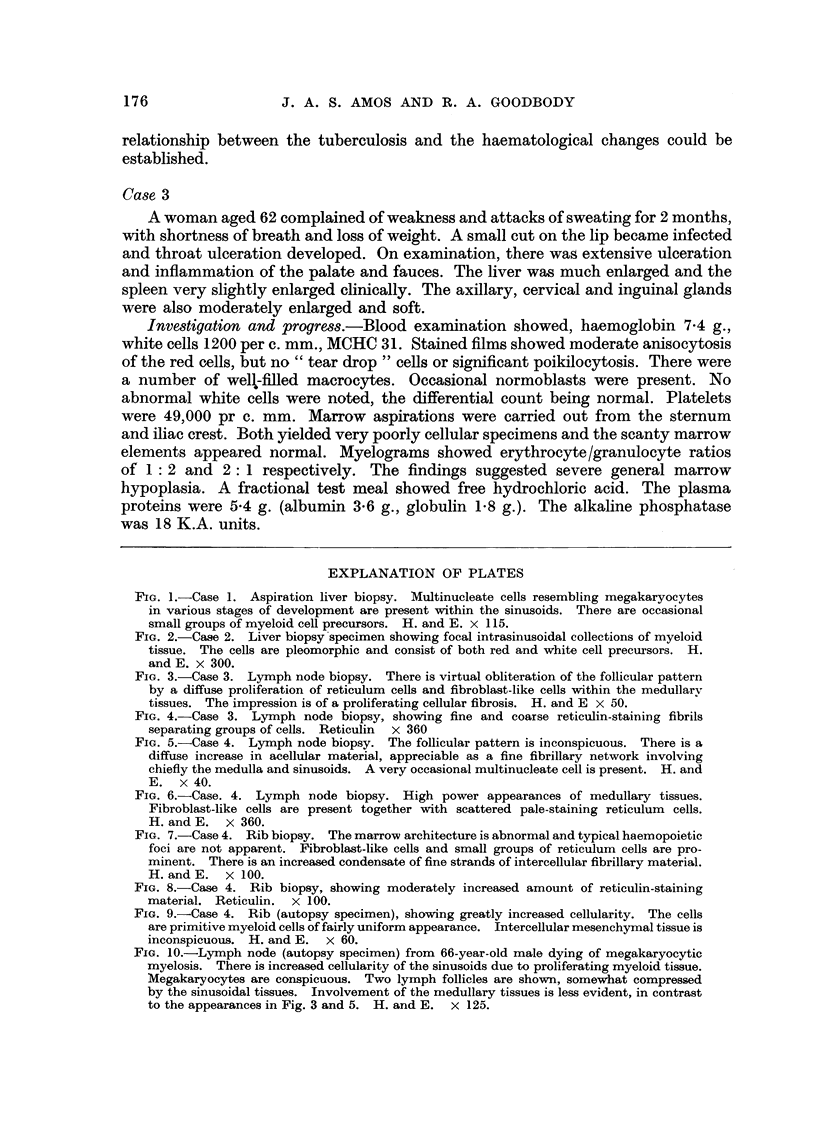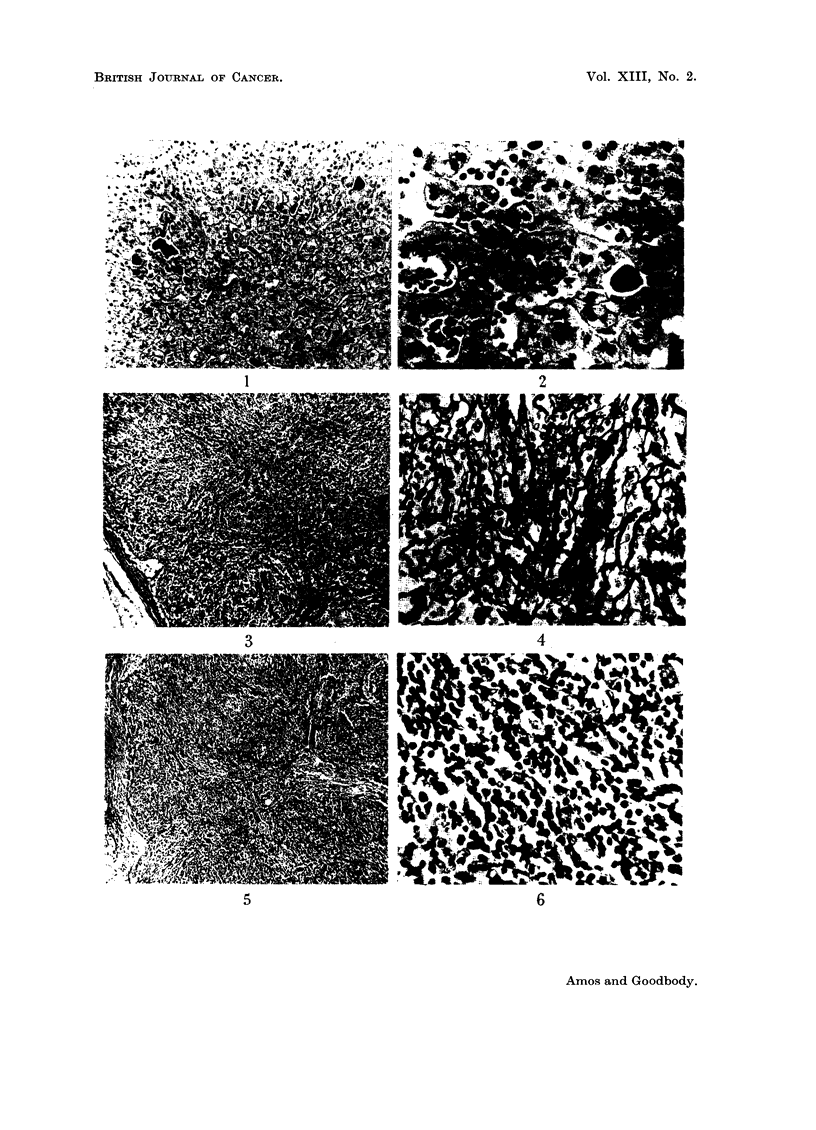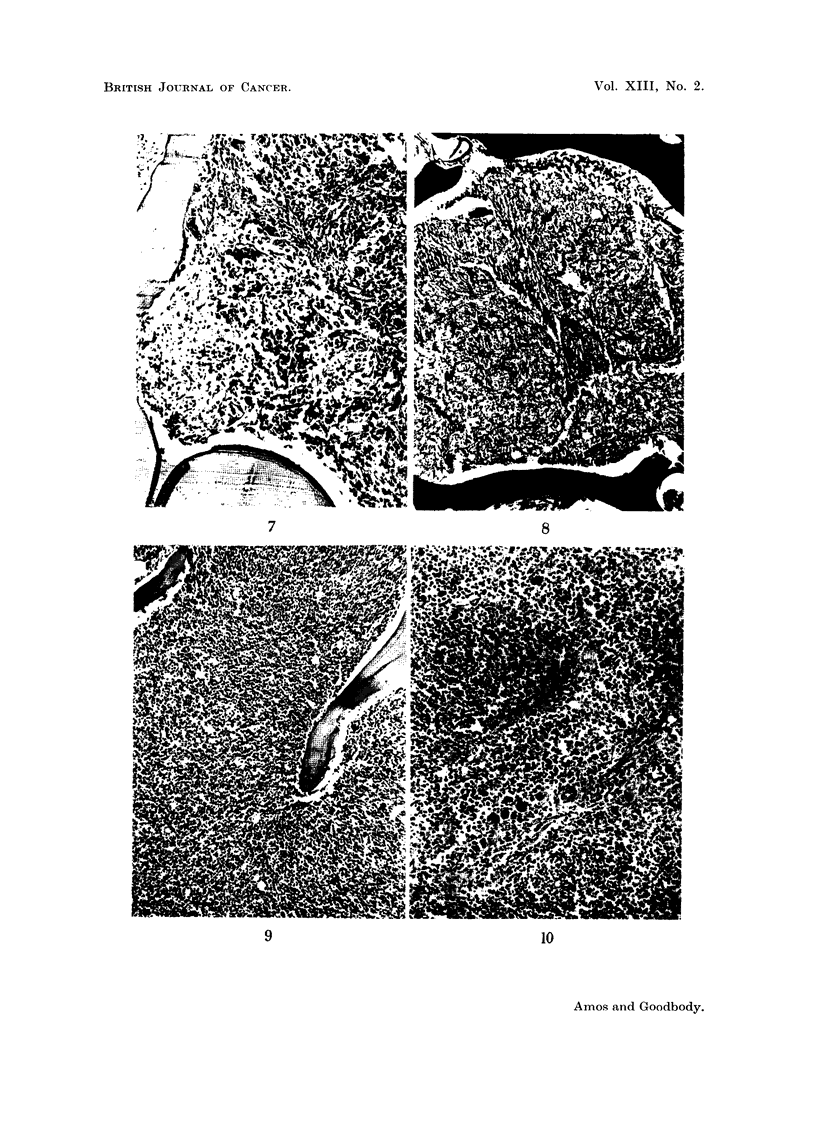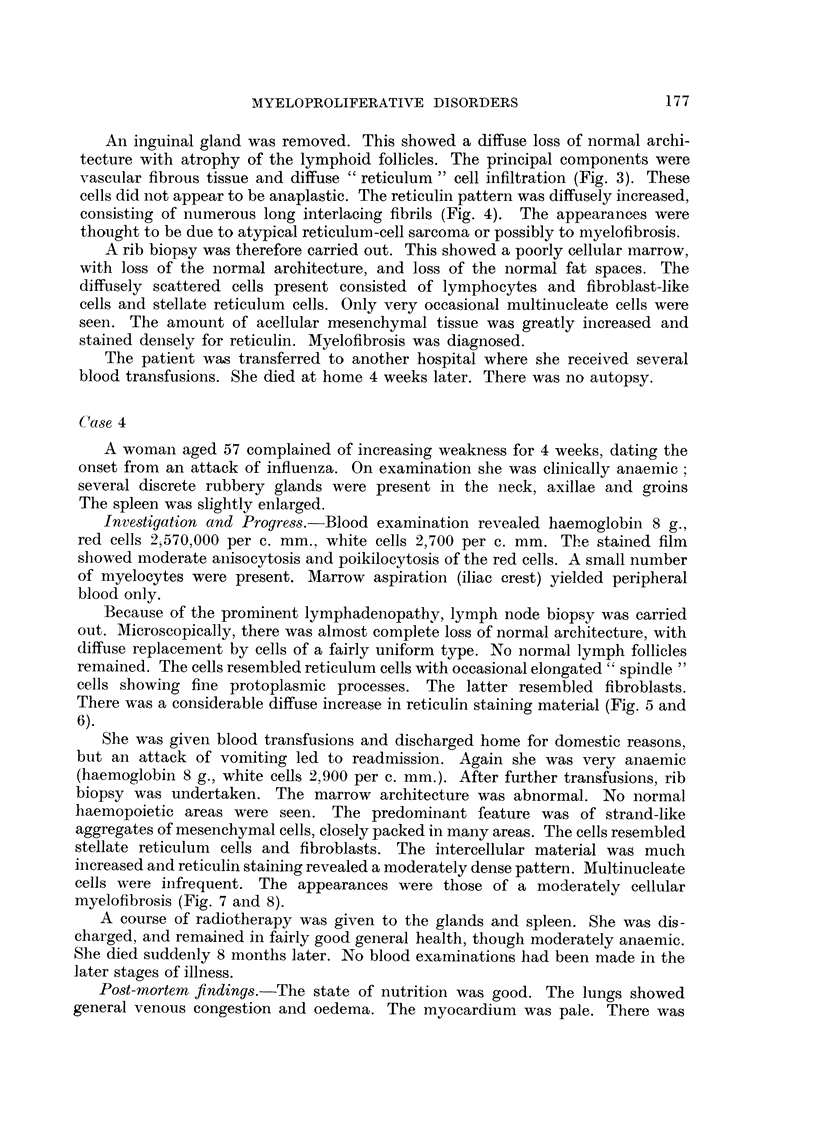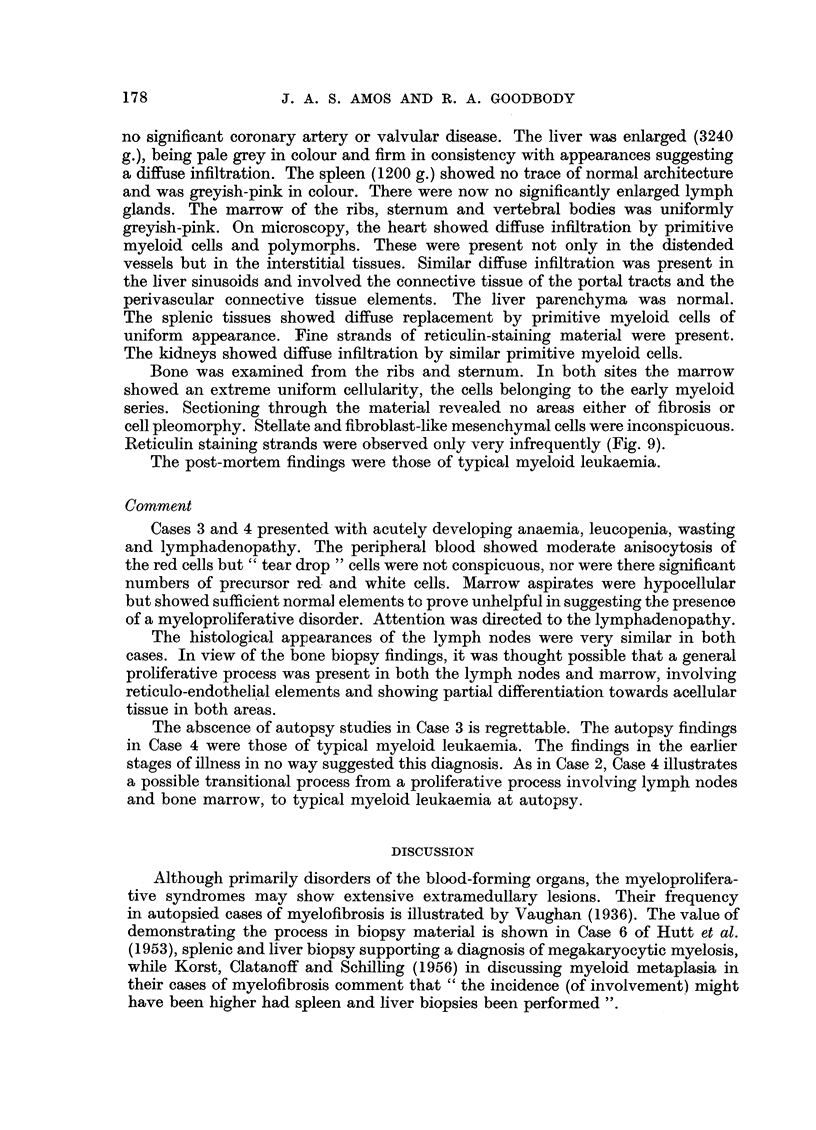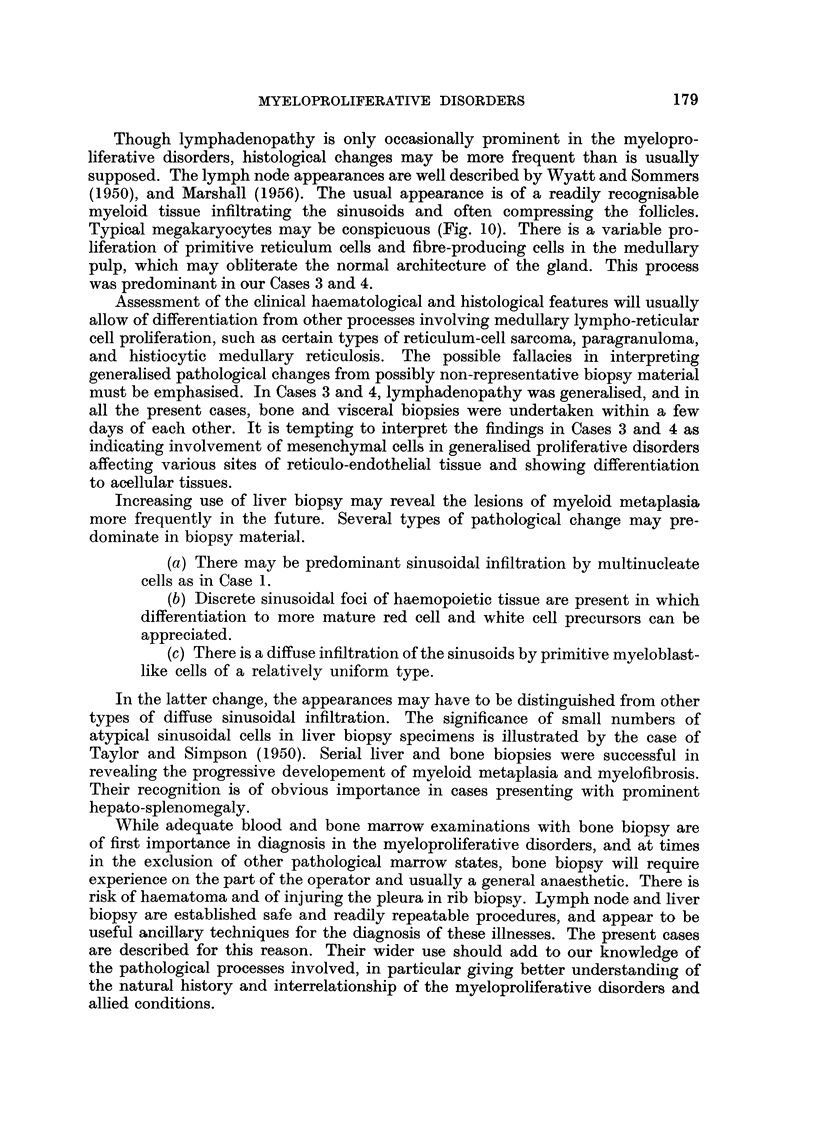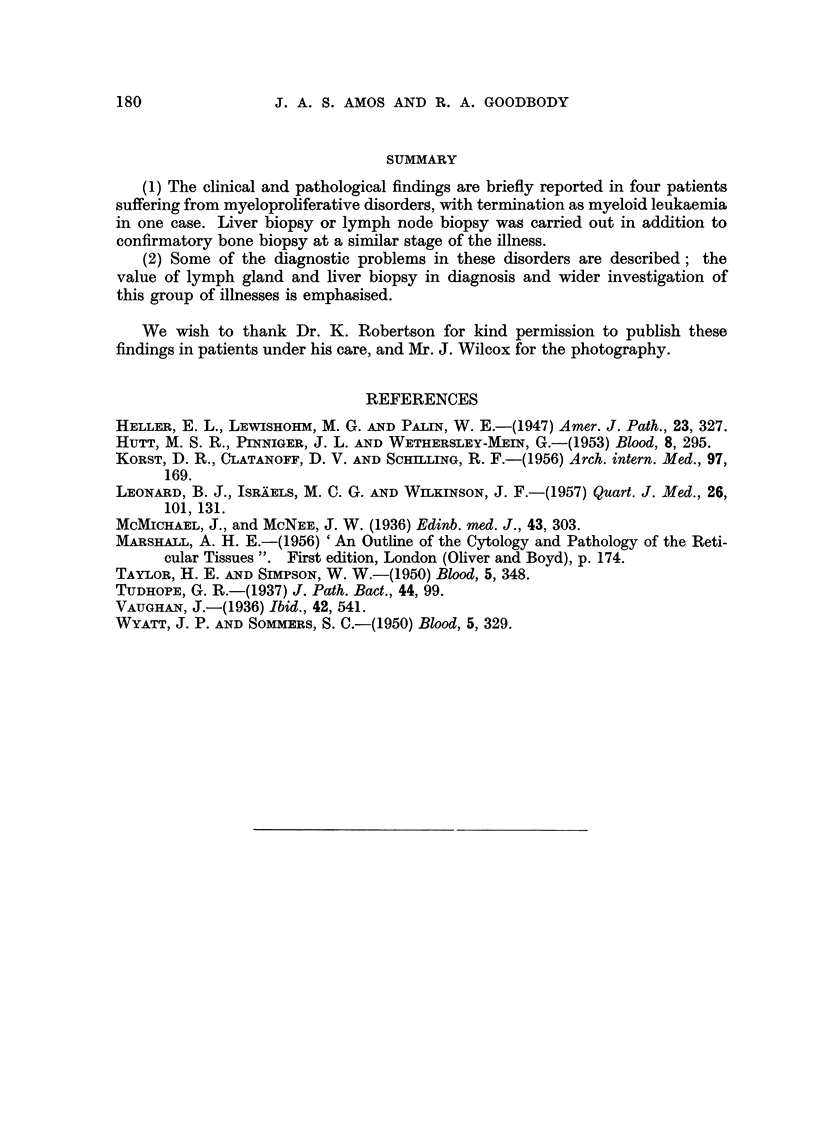# Lymph Node and Liver Biopsy in the Myeloproliferative Disorders

**DOI:** 10.1038/bjc.1959.24

**Published:** 1959-06

**Authors:** J. A. S. Amos, R. A. Goodbody

## Abstract

**Images:**


### LYMPH NODE AND LIVER BIOPSY IN THE MYELOPROLIFERATIVE DISORDERS

J. A. S. AMOS AND R. A. GOODBODY

*St. George's Hospital Medical School, Hyde Park Corner, London, S.W.1, and the Royal South Hants Hospital, Southampton*

Received for publication April 9, 1959

THE syndrome of anaemia, splenomegaly, leuco-erythroblastosis of the peripheral blood and hypocellularity of bone marrow aspirates is a well-recognised entity. The importance of bone biopsy in establishing a diagnosis of myelofibrosis in a number of these cases has been shown in recently reported case series and without it this diagnosis cannot be fully proven (Hutt, Pinniger and Wetherley-Mein, 1953 ; Korst, Clatanoff and Schilling, 1956). However, this condition or a related myeloproliferative disorder must be seriously suspected before a bone biopsy is decided upon and the very variable clinical presentation of the syndrome frequently results in other diagnoses being made first.

The splenic enlargement may be only slight and loss of weight and weakness are often the only symptoms, the clinical picture suggesting malignant cachexia. Lymphadenopathy is occasionally a presenting feature, and a primary disorder of lymph nodes may be suspected. In the peripheral blood, precursor cells of the red and white cell series may not be found (Tudhope, 1937), particularly in the early stages (Taylor and Simpson, 1950) or they may be present intermittently (McMichael and Mcnee, 1936). Anaemia is not necessarily severe. Some cases may present as a haemolytic anaemia or as a haemorrhagic disorder with platelet abnormalities. The findings on bone marrow aspiration are frequently equivocal and a proportion of patients yield a moderately cellular specimen showing normal marrow elements or prominent normoblastic red cell maturation (Leonard, Israëls and Wilkinson, 1956). Such appearances may draw attention away from the diagnosis of myelofibrosis or related disorder and the danger of drawing firm conclusions from a single marrow aspirate has been stressed (Heller, Lewisoh and Palin, 1947). Finally, prominent hepatomegaly or abnormalities of liver function may be the principal clinical findings and primary liver diseases may be suspected. The coexistence of cirrhosis and myelofibrosis is discussed by Wyatt and Sommers, (1950).

Initial diagnosis such as chronic myeloid leukaemia, " refractory " anaemia, or " malignant reticulosis " may result in therapy such as irradiation which may be harmful to the patient. When dealing with this obscure clinical group, any finding which suggests the possibility of a myeloproliferative disorder and indicates the need for bone biopsy examination will therefore be of importance. In a series of 20 cases of proven myelofibrosis or related disorder, four cases showed changes in either the lymph glands or the liver biopsy specimen which have helped in establishing the correct diagnosis. In all cases the diagnosis was finally confirmed by bone biopsy or autopsy studies. Their clinical and pathological features will be briefly discussed.

### Case Reports

*Case* 1

A woman of 30 suffered from occasional attacks of grand mal and had been treated by phenobarbitone and hydantoinates for several years. For two years she had been troubled by occasional transient dragging pains in the left flank, sometimes associated with sweating. In childhood she had rheumatic fever and chorea. On admission for investigation she was pale, the signs of mitral stenosis were present, the spleen was enlarged almost to the level of the umbilicus and the liver was just palpable.

*Investigations.*—A blood count showed haemoglobin 12·5 g., white cells 5,700 per c. mm. with normal differential count. The bleeding time, clotting time and platelet count were normal. The red cells showed considerable anisocytosis with an occasional macrocyte. Investigation of the renal tract showed no abnormalities. Marrow aspiration yielded a poorly cellular specimen. Scanty normal marrow elements were present.

The findings were suggestive of myelofibrosis and rib biopsy was carried out. As splintering occurred during removal it was thought that this material might prove unsatisfactory, so liver biopsy was undertaken a few days later. The liver biopsy showed many multinucleate cells within the sinusoids (Fig. 1). These cells had a pale homogenous acidophilic cytoplasm; some resembled mature megakaryocytes. A very occasional primitive myeloblast-like cell was present within the sinusoids. The bony material showed several areas of marrow. These contained groups of primitive myeloid cells among which were a number of bizarre megakaryocyte-like cells. Reticulum cells and fibroblast-like cells were present, and there was a moderate diffuse increase in reticulin. No normal erythropoietic tissue was seen, nor was there leucopoietic differentiation. The appearances were those of myelofibrosis.

*Progress.*—The abdominal pains were attributed to the splenic enlargement. She improved clinically and was discharged to another district, the diagnosis being " megakaryocytic myelosis and early myelofibrosis ". She died suddenly at home shortly afterwards and no autopsy was obtained.

*Case* 2

A man of 52 had complained for 7 years of occasional pains in the abdomen after meals, and bouts of vomiting. Gastric ulceration was demonstrated radiologically. He had been a coal miner for 21 years, and chest X-rays revealed diffuse reticulation with early lung fibrosis. He had recently complained of pains at the left costal margin, and examination showed the spleen to be just palpable. On admission to hospital for investigation of the splenomegaly, the blood pressure was 120/80 mm. Hg.

*Investigations.*—A blood count showed haemoglobin 16·6 g., red cells 6,040,000 per c. mm., white cells 26,100 per c. mm. The blood film revealed considerable polychromasia of the red cells with an occasional myelocyte and normoblast. Sternal marrow aspiration yielded an active cellular marrow, with hyperplasia of all marrow elements. A diagnosis of polycythaemia vera was made.

*Progress.*—During the next 5 years his abdominal symptoms were well controlled by symptomatic measures. He then complained of generalised pruritus and of impotence. The splenomegaly became more obvious, and he began to lose weight. He was paler, and dyspnoeic on exertion. Attacks of bronchitis with purulent sputum were frequent. Blood counts during this period showed an initial increase in values (haemoglobin 20·1 g., red cells 6,550,000 per c. mm.) followed by a steady fall in haemoglobin to 11·8 g. and 9·8 g. The white cell counts varied from 18,000 to 42,000 per c. mm. with small numbers of metamyelocytes (1 to 7 per cent) and of normoblasts. The platelet count remained normal. He received no irradiation or other specific treatment during the polycythaemic phase of illness.

Marrow aspirations 1, 3 and 5 years after his first admission yielded poorly cellular specimens, that at three years showing normal elements with a preponderance of cells of the myeloid series (myeloid : erythroid ratio, 8 : 1), while that at 5 years consisted almost entirely of peripheral blood.

Serial chest X-rays showed gradually increasing density of shadowing of the lung fields. Recent enlargement of the liver was noted. The development of hepatomegaly and of marrow failure following his previously polycythaemic state led to rib biopsy and liver biopsy.

Rib biopsy showed a moderately cellular marrow, the predominant cells resembling primitive myeloblasts. Some developing forms and polymorphs were present. Very many megakaryocytes in various stages of maturation were present. Erythropoiesis was inconspicuous. The reticulin pattern showed a general diffuse increase with focal areas of more closely enmeshed fibrils. Liver biopsy showed essentially normal parenchyma. The sinusoids contained diffuse and focal areas of myeloid tissue. A few of the focal collections showed the pleomorphic appearances of haemopoietic tissue with normoblastic development. The diffusely distributed cells were largely primitive myeloid cells, multinucleate cells being inconspicuous (Fig. 2).

He developed a persistent sinus of the chest wall, the discharge from which yielded tubercle bacilli on culture. Repeated sputum examinations throughout his illness had been negative for tuberculosis. He was discharged and died suddenly at home soon afterwards, and no autopsy was performed.

*Comment*

In Case 1 the chief symptom of left-sided abdominal pains was attributed to the enlarging spleen. The myeloproliferative process had been otherwise symptomless. Suggestive findings were the moderate anisocytosis of the red cells despite the reasonably high level of haemoglobin, and the poorly cellular marrow aspirate. Liver biopsy established the presence of megakaryocytic myelosis, bone biopsy confirming the presence of myelofibrosis.

Case 2 showed clinical and haematological evidence of polycythaemia vera, began to lose weight and became anaemic. He received no radiotherapy or other specific treatment. The liver became enlarged. Developing marrow failure was suspected and serial marrow aspirations supported this view. Rib biopsy was carried out to confirm this diagnosis, liver biopsy providing additional information as to the nature of the hepatomegaly and the presence of visceral involvement in the myeloproliferative process.

The case illustrates the transition from a polycythaemic process to myelofibrosis with myeloid metaplasia in the absence of specific treatment ; the coexistence of a tuberculous sinus is a further point of interest ; however no aetiological relationship between the tuberculosis and the haematological changes could be established.

*Case* 3

A woman aged 62 complained of weakness and attacks of sweating for 2 months, with shortness of breath and loss of weight. A small cut on the lip became infected and throat ulceration developed. On examination, there was extensive ulceration and inflammation of the palate and fauces. The liver was much enlarged and the spleen very slightly enlarged clinically. The axillary, cervical and inguinal glands were also moderately enlarged and soft.

*Investigation and progress.*—Blood examination showed, haemoglobin 7·4 g., white cells 1200 per c. mm., MCHC 31. Stained films showed moderate anisocytosis of the red cells, but no " tear drop " cells or significant poikilocytosis. There were a number of well-filled macrocytes. Occasional normoblasts were present. No abnormal white cells were noted, the differential count being normal. Platelets were 49,000 pr c. mm. Marrow aspirations were carried out from the sternum and iliac crest. Both yielded very poorly cellular specimens and the scanty marrow elements appeared normal. Myelograms showed erythrocyte/granulocyte ratios of 1 : 2 and 2 : 1 respectively. The findings suggested severe general marrow hypoplasia. A fractional test meal showed free hydrochloric acid. The plasma proteins were 5·4 g. (albumin 3·6 g., globulin 1·8 g.). The alkaline phosphatase was 18 K.A. units.

---

EXPLANATION OF PLATES

FIG. 1.—Case 1. Aspiration liver biopsy. Multinucleate cells resembling megakaryocytes in various stages of development are present within the sinusoids. There are occasional small groups of myeloid cell precursors. H. and E. × 115.

FIG. 2.—Case 2. Liver biopsy specimen showing focal intrasinusoidal collections of myeloid tissue. The cells are pleomorphic and consist of both red and white cell precursors. H. and E. × 300.

FIG. 3.—Case 3. Lymph node biopsy. There is virtual obliteration of the follicular pattern by a diffuse proliferation of reticulum cells and fibroblast-like cells within the medullary tissues. The impression is of a proliferating cellular fibrosis. H. and E × 50.

FIG. 4.—Case 3. Lymph node biopsy, showing fine and coarse reticulin-staining fibrils separating groups of cells. Reticulin × 360

FIG. 5.—Case 4. Lymph node biopsy. The follicular pattern is inconspicuous. There is a diffuse increase in acellular material, appreciable as a fine fibrillary network involving chiefly the medulla and sinusoids. A very occasional multinucleate cell is present. H. and E. × 40.

FIG. 6.—Case. 4. Lymph node biopsy. High power appearances of medullary tissues. Fibroblast-like cells are present together with scattered pale-staining reticulum cells. H. and E. × 360.

FIG. 7.—Case 4. Rib biopsy. The marrow architecture is abnormal and typical haemopoietic foci are not apparent. Fibroblast-like cells and small groups of reticulum cells are prominent. There is an increased condensate of fine strands of intercellular fibrillary material. H. and E. × 100.

FIG. 8.—Case 4. Rib biopsy, showing moderately increased amount of reticulin-staining material. Reticulin. × 100.

FIG. 9.—Case 4. Rib (autopsy specimen), showing greatly increased cellularity. The cells are primitive myeloid cells of fairly uniform appearance. Intercellular mesenchymal tissue is inconspicuous. H. and E. × 60.

FIG. 10.—Lymph node (autopsy specimen) from 66-year-old male dying of megakaryocytic myelosis. There is increased cellularity of the sinusoids due to proliferating myeloid tissue. Megakaryocytes are conspicuous. Two lymph follicles are shown, somewhat compressed by the sinusoidal tissues. Involvement of the medullary tissues is less evident, in contrast to the appearances in Fig. 3 and 5. H. and E. × 125.

1

2

3

4

5

6

Amos and Goodbody.

7

8

9

10

Amos and Goodbody.

An inguinal gland was removed. This showed a diffuse loss of normal architecture with atrophy of the lymphoid follicles. The principal components were vascular fibrous tissue and diffuse " reticulum " cell infiltration (Fig. 3). These cells did not appear to be anaplastic. The reticulin pattern was diffusely increased, consisting of numerous long interlacing fibrils (Fig. 4). The appearances were thought to be due to atypical reticulum-cell sarcoma or possibly to myelofibrosis.

A rib biopsy was therefore carried out. This showed a poorly cellular marrow, with loss of the normal architecture, and loss of the normal fat spaces. The diffusely scattered cells present consisted of lymphocytes and fibroblast-like cells and stellate reticulum cells. Only very occasional multinucleate cells were seen. The amount of acellular mesenchymal tissue was greatly increased and stained densely for reticulin. Myelofibrosis was diagnosed.

The patient was transferred to another hospital where she received several blood transfusions. She died at home 4 weeks later. There was no autopsy.

*Case* 4

A woman aged 57 complained of increasing weakness for 4 weeks, dating the onset from an attack of influenza. On examination she was clinically anaemic ; several discrete rubbery glands were present in the neck, axillae and groins The spleen was slightly enlarged.

*Investigation and Progress.*—Blood examination revealed haemoglobin 8 g., red cells 2,570,000 per c. mm., white cells 2,700 per c. mm. The stained film showed moderate anisocytosis and poikilocytosis of the red cells. A small number of myelocytes were present. Marrow aspiration (iliac crest) yielded peripheral blood only.

Because of the prominent lymphadenopathy, lymph node biopsy was carried out. Microscopically, there was almost complete loss of normal architecture, with diffuse replacement by cells of a fairly uniform type. No normal lymph follicles remained. The cells resembled reticulum cells with occasional elongated " spindle " cells showing fine protoplasmic processes. The latter resembled fibroblasts. There was a considerable diffuse increase in reticulin staining material (Fig. 5 and 6).

She was given blood transfusions and discharged home for domestic reasons, but an attack of vomiting led to readmission. Again she was very anaemic (haemoglobin 8 g., white cells 2,900 per c. mm.). After further transfusions, rib biopsy was undertaken. The marrow architecture was abnormal. No normal haemopoietic areas were seen. The predominant feature was of strand-like aggregates of mesenchymal cells, closely packed in many areas. The cells resembled stellate reticulum cells and fibroblasts. The intercellular material was much increased and reticulin staining revealed a moderately dense pattern. Multinucleate cells were infrequent. The appearances were those of a moderately cellular myelofibrosis (Fig. 7 and 8).

A course of radiotherapy was given to the glands and spleen. She was discharged, and remained in fairly good general health, though moderately anaemic. She died suddenly 8 months later. No blood examinations had been made in the later stages of illness.

*Post-mortem findings.*—The state of nutrition was good. The lungs showed general venous congestion and oedema. The myocardium was pale. There was no significant coronary artery or valvular disease. The liver was enlarged (3240 g.), being pale grey in colour and firm in consistency with appearances suggesting a diffuse infiltration. The spleen (1200 g.) showed no trace of normal architecture and was greyish-pink in colour. There were now no significantly enlarged lymph glands. The marrow of the ribs, sternum and vertebral bodies was uniformly greyish-pink. On microscopy, the heart showed diffuse infiltration by primitive myeloid cells and polymorphs. These were present not only in the distended vessels but in the interstitial tissues. Similar diffuse infiltration was present in the liver sinusoids and involved the connective tissue of the portal tracts and the perivascular connective tissue elements. The liver parenchyma was normal. The splenic tissues showed diffuse replacement by primitive myeloid cells of uniform appearance. Fine strands of reticulin-staining material were present. The kidneys showed diffuse infiltration by similar primitive myeloid cells.

Bone was examined from the ribs and sternum. In both sites the marrow showed an extreme uniform cellularity, the cells belonging to the early myeloid series. Sectioning through the material revealed no areas either of fibrosis or cell pleomorphy. Stellate and fibroblast-like mesenchymal cells were inconspicuous. Reticulin staining strands were observed only very infrequently (Fig. 9).

The post-mortem findings were those of typical myeloid leukaemia.

*Comment*

Cases 3 and 4 presented with acutely developing anaemia, leucopenia, wasting and lymphadenopathy. The peripheral blood showed moderate anisocytosis of the red cells but " tear drop " cells were not conspicuous, nor were there significant numbers of precursor red and white cells. Marrow aspirates were hypocellular but showed sufficient normal elements to prove unhelpful in suggesting the presence of a myeloproliferative disorder. Attention was directed to the lymphadenopathy.

The histological appearances of the lymph nodes were very similar in both cases. In view of the bone biopsy findings, it was thought possible that a general proliferative process was present in both the lymph nodes and marrow, involving reticulo-endothelial elements and showing partial differentiation towards acellular tissue in both areas.

The abscence of autopsy studies in Case 3 is regrettable. The autopsy findings in Case 4 were those of typical myeloid leukaemia. The findings in the earlier stages of illness in no way suggested this diagnosis. As in Case 2, Case 4 illustrates a possible transitional process from a proliferative process involving lymph nodes and bone marrow, to typical myeloid leukaemia at autopsy.

### DISCUSSION

Although primarily disorders of the blood-forming organs, the myeloproliferative syndromes may show extensive extramedullary lesions. Their frequency in autopsied cases of myelofibrosis is illustrated by Vaughan (1936). The value of demonstrating the process in biopsy material is shown in Case 6 of Hutt *et al.* (1953), splenic and liver biopsy supporting a diagnosis of megakaryocytic myelosis, while Korst, Clatanoff and Schilling (1956) in discussing myeloid metaplasia in their cases of myelofibrosis comment that " the incidence (of involvement) might have been higher had spleen and liver biopsies been performed ".

Though lymphadenopathy is only occasionally prominent in the myeloproliferative disorders, histological changes may be more frequent than is usually supposed. The lymph node appearances are well described by Wyatt and Sommers (1950), and Marshall (1956). The usual appearance is of a readily recognisable myeloid tissue infiltrating the sinusoids and often compressing the follicles. Typical megakaryocytes may be conspicuous (Fig. 10). There is a variable proliferation of primitive reticulum cells and fibre-producing cells in the medullary pulp, which may obliterate the normal architecture of the gland. This process was predominant in our Cases 3 and 4.

Assessment of the clinical haematological and histological features will usually allow of differentiation from other processes involving medullary lympho-reticular cell proliferation, such as certain types of reticulum-cell sarcoma, paragranuloma, and histiocytic medullary reticulosis. The possible fallacies in interpreting generalised pathological changes from possibly non-representative biopsy material must be emphasised. In Cases 3 and 4, lymphadenopathy was generalised, and in all the present cases, bone and visceral biopsies were undertaken within a few days of each other. It is tempting to interpret the findings in Cases 3 and 4 as indicating involvement of mesenchymal cells in generalised proliferative disorders affecting various sites of reticulo-endothelial tissue and showing differentiation to acellular tissues.

Increasing use of liver biopsy may reveal the lesions of myeloid metaplasia more frequently in the future. Several types of pathological change may predominate in biopsy material.

(*a*) There may be predominant sinusoidal infiltration by multinucleate cells as in Case 1.

(*b*) Discrete sinusoidal foci of haemopoietic tissue are present in which differentiation to more mature red cell and white cell precursors can be appreciated.

(*c*) There is a diffuse infiltration of the sinusoids by primitive myeloblast-like cells of a relatively uniform type.

In the latter change, the appearances may have to be distinguished from other types of diffuse sinusoidal infiltration. The significance of small numbers of atypical sinusoidal cells in liver biopsy specimens is illustrated by the case of Taylor and Simpson (1950). Serial liver and bone biopsies were successful in revealing the progressive developement of myeloid metaplasia and myelofibrosis. Their recognition is of obvious importance in cases presenting with prominent hepato-splenomegaly.

While adequate blood and bone marrow examinations with bone biopsy are of first importance in diagnosis in the myeloproliferative disorders, and at times in the exclusion of other pathological marrow states, bone biopsy will require experience on the part of the operator and usually a general anaesthetic. There is risk of haematoma and of injuring the pleura in rib biopsy. Lymph node and liver biopsy are established safe and readily repeatable procedures, and appear to be useful ancillary techniques for the diagnosis of these illnesses. The present cases are described for this reason. Their wider use should add to our knowledge of the pathological processes involved, in particular giving better understanding of the natural history and interrelationship of the myeloproliferative disorders and allied conditions.

SUMMARY

(1) The clinical and pathological findings are briefly reported in four patients suffering from myeloproliferative disorders, with termination as myeloid leukaemia in one case. Liver biopsy or lymph node biopsy was carried out in addition to confirmatory bone biopsy at a similar stage of the illness.

(2) Some of the diagnostic problems in these disorders are described; the value of lymph gland and liver biopsy in diagnosis and wider investigation of this group of illnesses is emphasised.

We wish to thank Dr. K. Robertson for kind permission to publish these findings in patients under his care, and Mr. J. Wilcox for the photography.

REFERENCES

Heller, E. L., Lewishohm, M. G. and Palin, W. E.—(1947) *Amer. J. Path.*, **23**, 327.
Hutt, M. S. R., Pinniger, J. L. and Wethersley-Mein, G.—(1953) *Blood*, **8**, 295.
Korst, D. R., Clatanoff, D. V. and Schilling, R. F.—(1956) *Arch. intern. Med.*, **97**, 169.
Leonard, B. J., Israëls, M. C. G. and Wilkinson, J. F.—(1957) *Quart. J. Med.*, **26**, 101, 131.
McMichael, J., and McNee, J. W. (1936) *Edinb. med. J.*, **43**, 303.
Marshall, A. H. E.—(1956) ' An Outline of the Cytology and Pathology of the Reticular Tissues ". First edition, London (Oliver and Boyd), p. 174.
Taylor, H. E. and Simpson, W. W.—(1950) *Blood*, **5**, 348.
Tudhope, G. R.—(1937) *J. Path. Bact.*, **44**, 99.
Vaughan, J.—(1936) *Ibid.*, **42**, 541.
Wyatt, J. P. and Sommers, S. C.—(1950) *Blood*, **5**, 329.